# Oxidative Fast Pyrolysis of High-Density Polyethylene on a Spent Fluid Catalytic Cracking Catalyst in a Fountain Confined Conical Spouted Bed Reactor

**DOI:** 10.1021/acssuschemeng.2c04552

**Published:** 2022-11-18

**Authors:** Santiago Orozco, Gartzen Lopez, Mayra Alejandra Suarez, Maite Artetxe, Jon Alvarez, Javier Bilbao, Martin Olazar

**Affiliations:** † Department of Chemical Engineering, University of the Basque Country UPV/EHU, P.O. Box 644, E48080Bilbao, Spain; ‡ IKERBASQUE, Basque Foundation for Science, 48009Bilbao, Spain; § Department of Chemical and Environmental Engineering, University of the Basque Country UPV/EHU, Nieves Cano 12, 01006Vitoria-Gasteiz, Spain

**Keywords:** Conical Spouted Bed Reactor, Waste Plastics, Oxidative Pyrolysis, FCC Catalyst, Catalytic Pyrolysis, Light Olefins

## Abstract

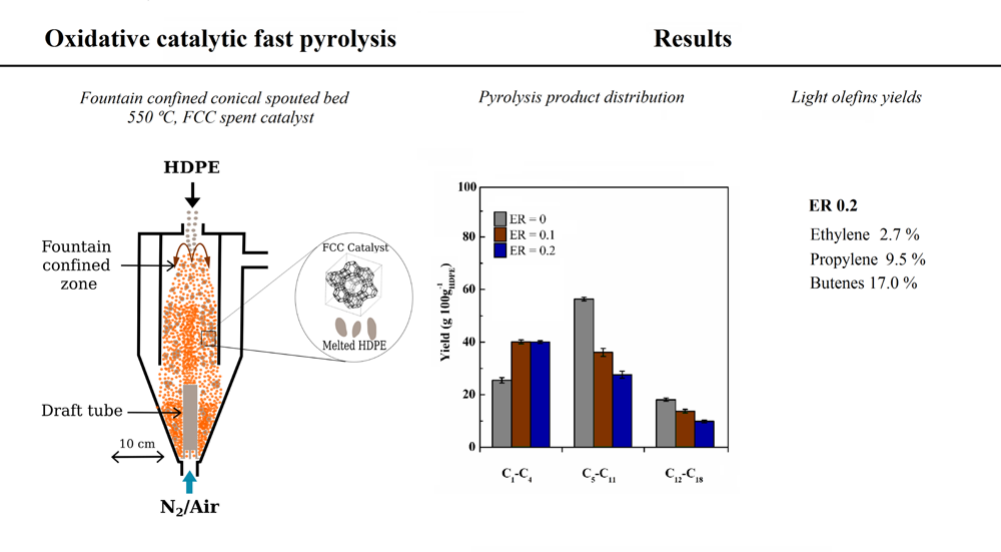

The oxidative fast pyrolysis of plastics was studied in a conical spouted bed reactor with a fountain confiner and draft tube. An inexpensive fluid catalytic cracking (FCC) spent catalyst was proposed for in situ catalytic cracking in order to narrow the product distribution obtained in thermal pyrolysis. Suitable equivalence ratio (ER) values required to attain autothermal operation were assessed in this study, i.e., 0.0, 0.1, and 0.2. The experiments were carried out in continuous regime at 550 °C and using a space-time of 15 g_catalyst_ min g_HDPE_^–1^. The influence of an oxygen presence in the pyrolysis reactor was analyzed in detail, with special focus on product yields and their compositions. Operation under oxidative pyrolysis conditions remarkably improved the FCC catalyst performance, as it enhanced the production of gaseous products, especially light olefins, whose yields increased from 18% under conventional pyrolysis (ER = 0) to 30% under oxidative conditions (ER = 0.1 and 0.2). Thus, conventional catalytic pyrolysis led mainly to the gasoline fraction, whereas light olefins were the prevailing products in oxidative pyrolysis. Moreover, the oxygen presence in the pyrolysis reactor contributed to reducing the heavy oil fraction yield by 46%. The proposed strategy is of great relevance for the development of this process, given that, on one hand, oxygen cofeeding allows solving the heat supply to the reactor, and on the other hand, product distribution and reactor throughput are improved.

## Abstract 2

A novel pyrolysis strategy is proposed for the efficient valorization of waste plastics using an inexpensive catalyst under autothermal conditions.

## Introduction

The suitable properties and flexibilities of plastic materials are promoting their leading role in several market sectors, such as packaging, automotive, building, or electronics. Thus, plastic production has monotonously increased in the last decades, reaching a global value of 36 million tons in 2020.^1^ Moreover, a substantial fraction of these plastics has a short life cycle and immediately ends in waste plastic streams.^2^ The unsuitable management of waste plastics and their nonbiodegradable natures cause several environmental problems associated with their accumulation in terrestrial and marine environments.^3,4^ According to recent estimations conducted in Europe in 2020, 23.4% of waste plastics had been sent to landfills, 42% used for energy recovery, and 34.6% recycled.^2^ According to the environmental impact of waste plastics and the underutilization of this valuable resource, the implementation of alternative recycling practices has become unavoidable in the present scenario.

Therefore, the development of feasible and sustainable waste plastic valorization routes is crucial. In the last decades, thermochemical processes, such as pyrolysis, gasification, and hydrothermal liquefaction, have undergone remarkable development.^5−8^ These processes allow converting waste plastics into valuable products, such as fuels, chemicals, and hydrogen, therefore promoting the development of a circular economy concept.^9^

Monomer recovery is the best alternative for a closed recycling loop of waste plastics. However, plastic thermal pyrolysis is not a selective process, and poor monomer productions have been reported.^8,10^ In fact, polystyrene is the only commodity plastic that leads to high monomer recoveries in thermal degradation, especially under fast pyrolysis conditions.^11,12^ Polyolefins are the plastics of highest production and therefore the main ones in waste plastic streams. Their thermal degradation takes place via a random radical scission mechanism, which leads to complex product distributions, including hydrocarbons from methane to solid waxes. Therefore, fast pyrolysis of polyolefins at moderate temperatures produces high wax yields and low gases and light oil.^13,14^ However, high temperatures and/or residence times shift the product distribution toward light compounds.^15,16^

The use of suitable catalysts for plastic pyrolysis has been regarded as a feasible alternative for the selective production of target valuable products.^8,17,18^ In this respect, different catalysts have been proposed for the production of fuels, light olefins, and aromatics from waste plastics.^19−26^ Zeolites are most commonly used in plastic pyrolysis due to their excellent properties. Thus, their high and tunable acidities together with shape selectivities allow for excellent control of products yields. Among the different zeolites with varying pore dimensions, structures, and acidities, HZSM-5 and HY are the most studied ones.^5,8^

Apart from the qualities of pyrolysis products, the full-scale development of waste plastic pyrolysis is conditioned by certain operational challenges, which are mainly associated with the low thermal conductivities and sticky natures of plastics, as well as the endothermic nature of the pyrolysis process. Therefore, a suitable reactor design is critical to guarantee plastic conversion under controlled and efficient conditions.^27^ Fluidized beds ensure isothermal operation and excellent gas–solid contact, which is essential in plastic catalytic pyrolysis.^28,29^ However, the performances of fluidized beds are conditioned by bed defluidization problems, which usually require operation with low values of the ratio of plastic feed rate to bed inventory.^30,31^ Conical spouted bed reactors are an alternative to conventional bubbling fluidized beds, as they are able to create vigorous solid circulation regimes and therefore attenuate bed defluidization problems.^32,33^ Moreover, the incorporation of a fountain confiner and draft tube helps the development of vigorous fluidization regimes with improved gas–solid contacts and solid circulations; i.e., they lead to an enhanced fountain regime.^34^ This novel reactor configuration was originally developed for biomass catalytic gasification processes and involves a step forward in terms of catalytic cracking efficiency,^35,36^ as it leads to significant reductions in tar yield.

The main aim of this study is to step forward in the challenges involving heat supply to the pyrolysis reactor in plastic pyrolysis. Thus, autothermal operation by cofeeding air with the fluidizing agent is proposed for the scaling up of this process.

In fact, as the scale of the process is larger, the heat supply to the pyrolysis reactor is more difficult.^37^ This is mainly because heat demand for plastic pyrolysis is closely related to their volume, whereas heat transfer is limited by the reactor surface.^38^ Moreover, the poor thermal conductivities of plastics may also condition the heat transfer inside the reactor. However, autothermal operation ensures a well distributed heat release within the whole reactor by the combustions of the plastic materials or their derived volatiles.

This strategy has been commonly applied in biomass pyrolysis.^38^ However, biomass oxidative pyrolysis studies are in an early stage, as they are commonly performed in small lab-scale reactors or thermogravimetric analyzers (TGA), in which the influence of oxygen on reaction kinetics is analyzed.^38^ In addition, some relevant studies have been reported in different reactor designs operating in a continuous regime. Among them, fluidized bed reactors are the most used ones,^39,40^ although fixed beds^41^ and spouted beds^42^ have also been proposed for biomass oxidative pyrolysis. In spite of the promising results obtained in the oxidative pyrolysis of biomass, i.e., significant improvements in bio-oil and char quality,^38^ this process has not been studied for plastic valorization. Accordingly, this paper deals with fast oxidative pyrolysis on a spent cracking catalyst for the valorization of waste plastics in a conical spouted bed reactor by operating in a continuous regime.

Finally, an additional advantage of an oxygen presence in the reaction environment is the in situ catalyst regeneration by coke combustion. In this paper, a spent FCC or equilibrium catalyst (E-cat) was used in situ. In spite of a lower activity than a fresh FCC catalyst due to the poisoning of active sites and zeolite dealumination,^20^ these catalysts have suitable activity for the cracking of waste plastics.^43−46^ Thus, high selectivity toward valuable chemicals, such as light olefins and aromatics, and a gasoline fraction have been reported when an E-cat was used for plastic conversion.^45,47,48^ Moreover, reuse of this inexpensive catalyst is highly relevant from an environmental perspective, as landfilling of this hazardous waste can be avoided. Accordingly, the autothemal catalytic fast pyrolysis of plastics on spent FCC catalysts was approached in this study by analyzing energy demand and the role played by an oxygen presence on product yields and their compositions.

## Experimental Section

### Catalyst

The catalyst selected for this study is a spent FCC catalyst from Petronor Refinery (Spain). In order to attain the optimum performance of this catalyst in the reactor, the whole amount was sieved to a particle size in the 90–150 μm range. The catalyst is made up of HY zeolite (16 wt %) and different additives, such as silica, alumina, and clay, which provide a suitable pore distribution and physical properties. The HY zeolite is characterized by its uniform pore channels of 7.4 Å × 7.4 Å, which ensure a shape selectivity allowing the diffusion of hydrocarbons with less than 12 carbon atoms.^49,50^ Given that the spent FCC catalyst was used for a long period of time in the refinery, it contains different metal oxides (Fe, MgO, NiO, Ca, Na_2_O, TiO_2_, MnO, P_2_O_5_, and V_2_O_5_).

The porous structure of the catalyst was analyzed by N_2_ adsorption–desorption (Micromeritics ASAP 2010). Moreover, the acid properties were assessed by NH_3_ adsorption–desorption. Thus, the values of total acidity and acid strength distribution were determined by means of temperature programed desorption of NH_3_ in an Autochem II 2920 instrument. The procedure was as follows: (i) removal of the adsorbed volatile impurities with a He stream following a ramp of 15 °C min^–1^ to 550 °C, (ii) adsorption of NH_3_ (750 μL min^–1^) until reaching sample saturation, (iii) desorption of the physisorbed NH_3_ with a He stream at 150 °C, and (iv) desorption of the chemisorbed NH_3_ at programmed temperature (5 °C min^–1^) from 150 to 550 °C, with the TCD signal being recorded continuously. The acid site distribution was established according to the following desorption levels: weak acidity, 150–250 °C; medium acidity, 250–400 °C; strong acidity, 400–550 °C. The catalyst characterization results are summarized in Table 1. As observed, the spent FCC catalyst is basically a microporous material with a BET surface area of 143 m^2^ g^–1^. In spite of the deterioration of the catalyst in the refinery, it has a reasonable value of total acidity (124 μmol_NH3_ g^–1^) with medium acid sites being the prevailing ones.

**Table 1:** Physical and Acid Properties of Spent FCC Catalyst Used

BET surface area (m^2^ g^–1^)	143
S_micropore_ (m^2^ g^–1^)	103
Average pore diameter (Å)	101
Pore volume distribution (%)	
<20; 20 < dp(Å) < 500; >500	5.7; 7.6; 86.7
Total acidity (μmol_NH3_ g^–1^)	124

### Experimental Equipment and Operating Conditions

The plastic used in this study is pure HDPE supplied by Dow Chemical Company (Tarragona). The HDPE was fed into the reactor as received, i.e., in the form of pellets with their average diameter being 4 mm. In fact, this size is especially suitable for the pyrolysis unit feeding system. The main properties of this polymer are as follows: average molecular weight, 46.2 kg mol^–1^; polydispersity, 2.89; density, 940 kg m^–3^; and higher heating value, 43 kJ kg^–1^. The latter was determined using an isoperibolic bomb calorimeter (Parr 1356).

**Figure 1:**
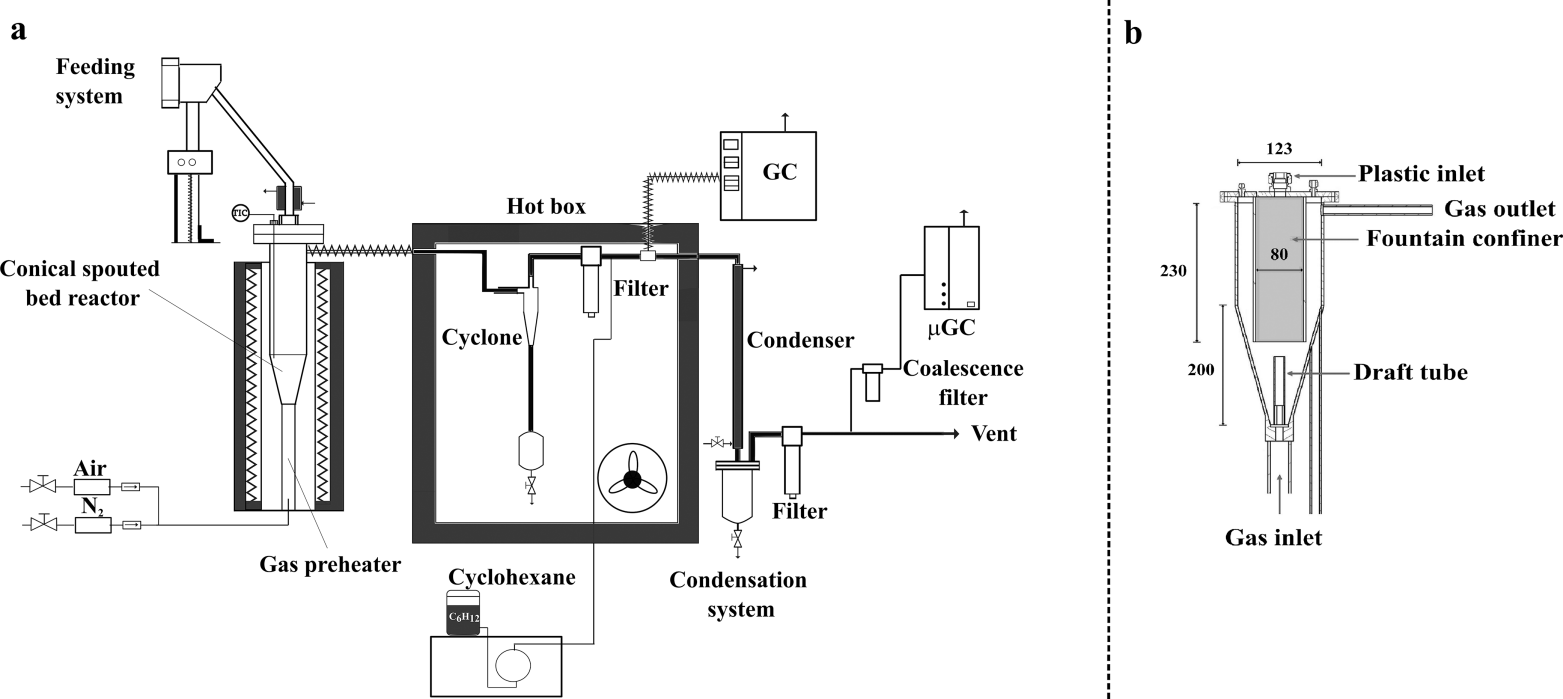
Scheme of the bench scale plant used for catalytic cracking of HDPE on the spent FCC catalyst (a) and of the fountain confined conical spouted bed reactor (dimensions in mm) (b).

The continuous fast pyrolysis experiments were conducted in a bench scale unit provided with a fountain confined conical spouted bed reactor; Figure 1a shows a scheme of this unit. The main element of the plant is the pyrolysis reactor, which is a conical spouted bed equipped with two internal devices to improve its hydrodynamic performance, i.e., a nonporous draft tube and a fountain confiner (Figure 1b). The detailed design and reactor dimensions can be found elsewhere.^51^ Although the conventional conical spouted bed performs well in the pyrolysis of biomass and other wastes,^52−54^ the use of the mentioned internals makes this reactor especially suitable for handling fine materials, such as FCC catalyst particles.^34^ On the one hand, the incorporation of a draft tube decreases the gas flow rate required and improves bed stability.^55,56^ On the other hand, the fountain confiner avoids fine particle elutriation and contributes to improving bed stability.^57^ Therefore, this reactor configuration allows operating under vigorous fluidization regimes, greatly improving the gas–solid contact, which is essential in catalytic processes. In fact, the fountain confined conical spouted bed reactor performs well in the catalytic gasification of biomass.^35,36,58^ It should be noted that the primary catalysts used for tar abatement in gasification process are of limited activity, and therefore, a highly efficient contact with the catalyst is required. Likewise, low activity spent FCC catalysts also require efficient contact to ensure full conversion of plastics to valuable products.

Pyrolysis runs were carried out in continuous regime by feeding 1 g min^–1^ of HDPE. The feeding system consisted of a vertical piston whose ascension speed determines the plastic feed rate. The top of the feeding device is connected to an inclined pipe that discharges the plastic into the reactor, specifically into the core of the fountain confiner (Figure 1b). The feeding device is equipped with a vibrator to ease polymer flow.

The plant is equipped with two mass flow controllers to measure nitrogen and air gas flow rates. In all cases, a total gas flow rate of 6.5 L min^–1^ was used, which corresponds to approximately 4 times the minimum spouting one. This gas flow rate allows operating under highly vigorous spouting conditions, i.e., enhanced fountain regime conditions. This regime is characterized by a high fountain, reaching the top of the confiner and ensuring vigorous solid circulation.^34^ In order to operate under the desired equivalence ration (ER), the nitrogen was partially replaced by air, with the total gas flow rate being maintained constant. The ER ratio was defined as the ratio between the actual air flow rate and the stoichiometric one required for full combustion of the plastic fed into the pyrolysis reactor. The suitable ER values to operate under autothermal conditions, i.e., without external heat input, are detailed in the Determination of ER Values for Autothermal Operation section. Under these conditions, the average gas residence time in the reactor is very low. Furthermore, there is a distribution of residence times due to the regions of different dilution in the reactor. Thus, the residence time in the spout is very short (in the order of 20 ms). This low value is associated with the high gas velocity and the small volume of this region (the draft tube inner volume). However, the gas flow rate crossing the annulus is lower, and its volume is higher; i.e., the low porosity in this zone leads to gas residence times in the order of 1 s.

The bed was a mixture of 135 g of sand (0.2–0.3 mm) and 15 g of FCC catalyst (90–150 μm), corresponding to a space-time value of 15 g_cat_ min g_HDPE_^–1^. Moreover, a temperature of 550 °C was selected to perform the experimental runs. These suitable conditions were stablished in a previous parametric study in which the influences of the main process conditions were determined under inert pyrolysis conditions.^44^ Thus, these conditions ensure full conversion of waxes to hydrocarbons in the C_1_–C_18_ range.

Prior to chromatographic analysis and condensation, the product stream leaving the reactor crossed a cyclone and sintered a steel filter (25 μm) in order to retain fine sand/catalyst particles that may entrain from the reactor. These elements are located inside an oven maintained at 300 °C to avoid the condensation of heavy pyrolysis products. The condensation system includes a double-shell tube condenser cooled with tap water and a coalescence filter to retain any aerosol drop from the gaseous stream prior to analysis.

The product stream was analyzed in-line by an Agilent 7890 chromatograph (GC) prior to its condensation. This GC is provided with a flame ionization detector (FID). The temperature program is as follows: (i) 4.5 min at 45 °C, (ii) ramp of 15 °C min^–1^ to 305 °C, and (iii) 5.5 min at 305 °C to ensure that all hydrocarbons were eluted from the HP-PONA column. The sample was transferred to the GC through a line thermostated at 230 °C to avoid the condensation of any heavy product. This temperature ensures suitable analyses of liquid hydrocarbons formed in plastic pyrolysis (C_5_–C_11_ and C_12_–C_18_ fractions) and avoids the entrance of waxes into the GC column. It should be pointed out that the conditions used in this study, high space-time and moderate temperature, favor full conversion of waxes to lighter products, but operation with lower values of the mentioned parameters lead to wax formation.^44^ Cyclohexane (not formed in the process) was used as an internal standard to attain the mass balance closure. This compound was fed into the product stream at the outlet of the sintered steel filter. In addition, the compositions of light gases (C_1_–C_4_ hydrocarbons, CO, and CO_2_) were determined in a G.A.S. Compact GC^4.0^ chromatograph, which is provided with two detectors (FID and TCD) and three columns (MXT-Q Bond, MXT-Msieve 5A, and RT-Q-Bond). The liquid compounds collected in the condensation system were identified by means of GC-MS (Shimadzu 2010-QP2010S) provided with a DB-1MS column. This procedure allowed detailed identification of the hydrocarbons in the pyrolysis oil. Given the low molecular weight of certain products in the catalytic pyrolysis of plastics, this stream was bubbled through cyclohexane to ease their collection for subsequent GC-MS analysis. The information gathered about gas and oil fractions by GC, Compact GC, and GC-MS was used for determining product yields and their compositions. The GC allows analyzing hydrocarbons from C_1_ to C_18_, but waxes cannot be determined in this device. Thus, the yields of waxes were obtained from the overall mass balance, once the amounts of gaseous and liquid hydrocarbons were obtained based on an internal standard (cyclohexane).

The GC and microGC analyses were performed subsequent to at least 10 min steady operation, and runs have been conducted at least five times under the same conditions to ensure reproducibility of the results. The experimental error under these conditions was below 4% in all cases.

## Results and Discussion

### Determination of ER Values for Autothermal Operation

The heat requirement of the process should be determined in order to fix the ER values required to attain autothermal operation. Thus, the heat required in the process is due to mainly three facts: (i) the energy required to heat the fluidizing gas to the pyrolysis temperature, (ii) the energy associated with heating, degradation, and devolatilization of the polymer, and (iii) energy losses. Accordingly, the energy produced in the partial combustion of the plastic must account for the mentioned requirements (eq 1).
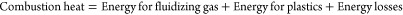
1

The heat released by combustion reactions was assumed to be only due to the combustion of HDPE and not to the pyrolysis volatiles (eq 2).
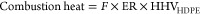
2where *F* is the mass flow rate of the plastic, ER the equivalence ratio, and HHV_HDPE_ the higher heating value of the polymer.

The energy required to heat the fluidizing gas may be determined based on the flow rate, composition, and change in temperature of the gaseous stream
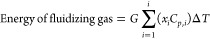
3where *G* is the gas flow rate, *x_i_* the mass fraction of each compound in the fluidizing gas stream, and *C_p,i_* their corresponding heat capacities. Although the temperature range considered is rather wide, from room temperature to 550 °C, it is reasonable to assume constant *C_p_* values. Thus, a nitrogen flow rate of 6.5 l min^–1^ was used under the studied conditions. Therefore, the energy required to heat this stream from room temperature to pyrolysis conditions (550 °C) is approximately 4360 J min^–1^.

However, estimations of the heat associated with plastic heating and pyrolysis are much more complex. Different theoretical and experimental approaches have been proposed in the literature for determining this heat. Furthermore, the estimations depend on pyrolysis conditions, especially heating rate.^59,60^ Extrapolation of the values determined under low heating rates to fast pyrolysis conditions is a challenging task. Stoliarov and Walters^61^ used differential scanning calorimetry (DSC) and determined experimentally the evolution of heat capacities with temperature for different polymers and the overall heats associated with the melting and degradation steps. Thus, in the case of HPDE, the overall heat requirement determined by these authors is 2510 J g^–1^. The methodology developed by Agarwal and Lattimer^62^ was also based on the DSC technique. However, these authors determined the heats associated with each step involved in the pyrolysis of several materials and estimated an overall heat of 1940.9 J g^–1^ for HDPE. Moreover, Staggs^63^ determined heats in the 1826–2981 J g^–1^ range. Considering the values reported in the recent literature, an average value of 2200 J g^–1^ was considered for polymer heating and degradation. Given that the plastic feed rate was 1 g min^–1^, the heat flow required is 2200 J min^–1^.

Accordingly, the overall heat requirement without considering heat losses is around 6500 J min^–1^, i.e., the addition of the heat required for gas stream heating and polymer heating and degradation. Assuming a combustion enthalpy of 43000 J g^–1^ for HDPE, the ER required to ensure autothermal operation is around 0.15; that is, 15% of the polymer feed must be burned to obtain the required 6500 J min^–1^. It is of note that heat losses were not included in this calculation because, unlike industrial reactors, insulation is not a design priority in laboratory- and bench-scale reactors, and therefore, heat losses in these units are high. Therefore, three ER values were analyzed in this study, i.e., 0.0, 0.1, and 0.2. These values correspond to oxygen concentrations of 0.0, 3.7, and 7.4 vol % in the fluidizing gas fed into the pyrolysis process.

It should be noted that the autothermal ER value determined is strongly conditioned by the scale of the plant. Thus, as the scale of the plant is larger, the ratio of the gas flow rate/plastic feed rate ratio is considerably lower, and therefore, the heat requirements per plastic mass unit fed into the reactor are lower. Furthermore, the heat integration of a larger pyrolysis unit is more efficient, thereby reducing the ER value required to attain an autothermal regime. In this respect, Amutio et al.^42^ reported a reduction in the autothermal ER ratio from 0.127 in a bench-scale unit to 0.073 in a 500 kg h^–1^ pilot plant. In addition, several authors determined ER values for autothermal operation in biomass pyrolysis, and their estimations range from 0.05 to 0.14,^39,40,42,64^ depending on the type of biomass and reactor design.

### Effect of ER on Product Yields and Their Compositions

#### Product Yields

**Figure 2:**
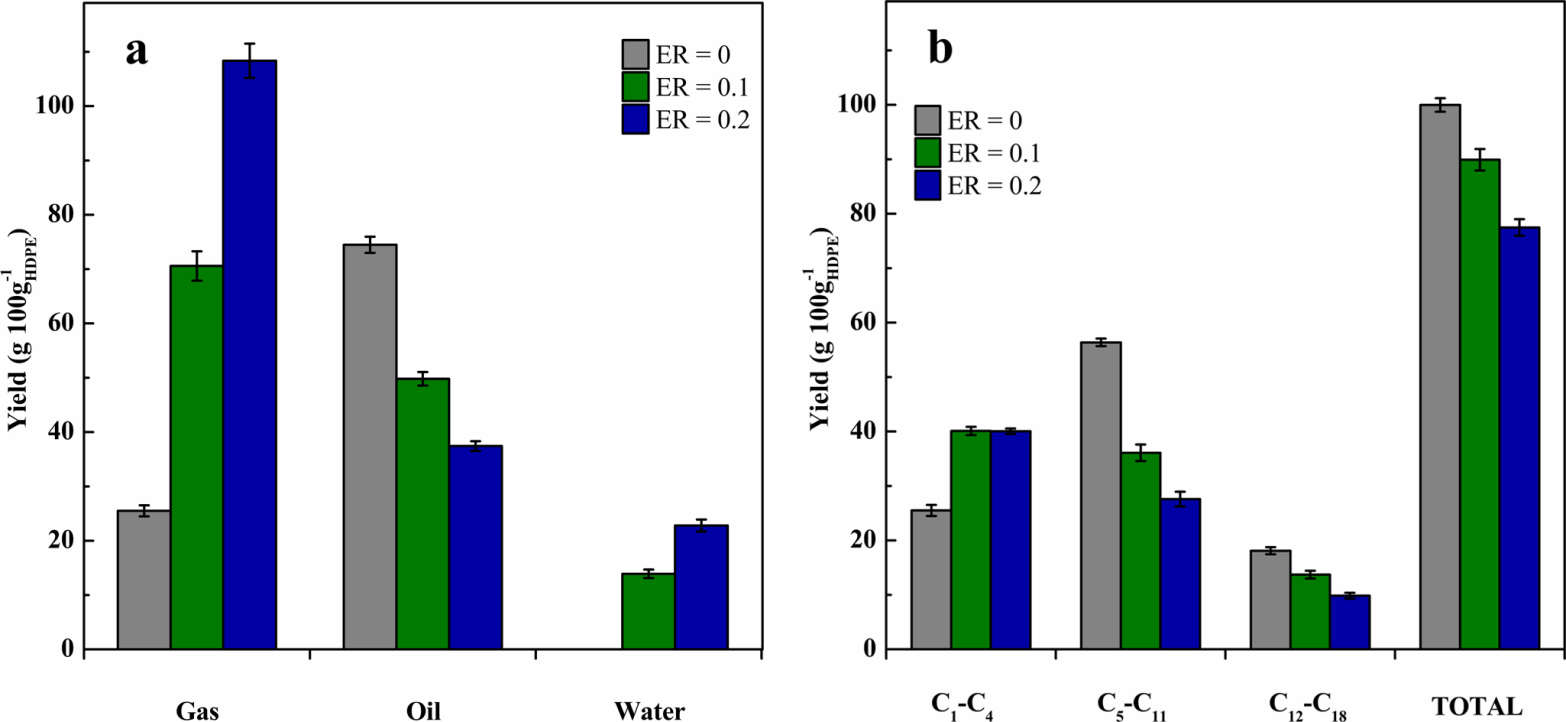
Product distribution obtained in the oxidative catalytic pyrolysis with different ER ratios at 550 °C on the FCC catalyst. All the products (a) and only hydrocarbons (b).

The influence of ER on the continuous oxidative catalytic pyrolysis of HDPE was studied at 550 °C. Thus, the results obtained in conventional pyrolysis (ER = 0) were compared with those obtained operating with ER values of 0.1 and 0.2. Product yields based on 100 g of plastic in the feed are shown in Figure 2a. As observed, oxygen incorporation into the products leads to yields higher than 100, which is because 34 and 69 g of oxygen were added to the products when the operation was carried out with ER values of 0.1 and 0.2, respectively. It should be noted that no significant oxygen concentration was detected in the pyrolysis gases. Accordingly, it was assumed that the oxygen in the feed was fully consumed in combustion reactions. As observed in Figure 2a, gas production greatly increased under oxidative catalytic pyrolysis of plastics, but the yields of the liquid fractions, both C_5_–C_11_ and C_12_–C_18_ fractions, decreased. It is noteworthy that no waxes were detected given that moderate temperature and high space-time were used in this study. This result evidenced the suitable performance of the combination of the spent FCC catalyst and the fountain confined conical spouted bed reactor for the cracking of waxes, which are primary products in the thermal degradation of polyolefins and the main ones under fast pyrolysis conditions at low or moderate temperatures.^14,65−67^

Moreover, combustion reactions also lead to water formation, whose yield increased almost proportionally with the oxygen flow rate in the feed. Furthermore, the higher gas yields reported under oxidative pyrolysis are also associated with CO and CO_2_ formations. Thus, around 30% of the oxygen fed into the pyrolysis reactor was detected in the products accounting for water, with this value being slightly lower for the higher ER value used. The remaining oxygen was transformed into CO_2_ (60% of the oxygen in the feed) and CO (between 2.4% and 4% depending on the ER ratio used). Hydrogen distribution in the pyrolysis products greatly depends on the ER used. Thus, an increase in ER from 0.1 to 0.2 promoted hydrogen recovery in the water from the product stream (from 10.7% to 17.6%, respectively), with the remaining hydrogen being in the hydrocarbons. The recovery of carbon in the hydrocarbons decreased when operating under oxidative conditions, but the recovery by forming CO and CO_2_ increased. Thus, the latter increased from 10.0% to 22.6% when the ER ratio was increased from 0.1 to 0.2. Therefore, it may be concluded that the decrease in the liquid hydrocarbons is related to their partial oxidation to yield gases (CO and CO_2_) and water. However, a more detailed evaluation of the yields of hydrocarbons (Figure 2b) clearly reveals the role played by an oxygen presence in the reaction environment. Thus, oxidative conditions lead to a significant increase in the yields of gaseous C_1_–C_4_ hydrocarbons, whereas light oil (C_5_–C_11_) and heavy oil (C_12_–C_18_) productions were remarkably reduced. Considering the fact that oxidation of noncondensable gaseous products is faster than oxidation of condensable ones (oil fraction), and therefore are presumably the main components burned, the differences observed may be associated with a different cracking mechanism under oxidative conditions. It should be noted that this kind of catalyst is prone to suffer a quick deactivation, as has been widely reported in FCC industrial units, in which activity decay is remarkable after only a few seconds of residence time in the riser.^68−71^ The situation is not comparable to that attained in the pyrolysis of plastics due to the much higher catalyst/oil ratio used in the latter and the different composition of the plastic-derived volatile stream. However, a quick deactivation of the FCC catalyst may also be expected. Therefore, an oxygen presence seems to promote in situ combustion of the coke deposited on the FCC catalyst, which leads to its partial regeneration and so enhances catalyst activity throughout continuous catalytic pyrolysis. Accordingly, cracking reactions to convert liquid hydrocarbons into gaseous products were enhanced as the ER ratio was raised. This result is highly relevant, as oxidation reactions affect mainly gaseous products. There was also an increase in gaseous hydrocarbons, which is clear evidence of the significant improvement in the catalyst cracking activity. Overall, there is an increase in the selectivity toward valuable products, such as light olefins, as well as in the reactor throughput due to the higher catalyst cracking activity.

The aforementioned results clearly show a higher conversion of pyrolysis products toward lighter compounds when ER is increased, which is associated with the increase in the activity of the FCC catalyst due to the in situ regeneration of its active sites. In previous studies, Olazar et al.^72^ compared the performance of the fresh FCC catalyst and those subject to steaming treatments, namely, mild conditions (5 h at 760 °C) and severe ones (8 h at 816 °C). Thus, an increase in the steaming duration and temperature caused a reduction in catalyst acidity, which led to higher yields of the heavier oil fraction together with a remarkable reduction in C_1_–C_4_ gaseous hydrocarbons. Likewise, Ali et al.^73^ studied catalytic pyrolysis of plastics in a fluidized bed reactor on fresh and equilibrated FCC catalysts, and the acidity reduction after catalyst steaming shifted the product distribution from C_1_–C_4_ toward liquid hydrocarbons. Moreover, other authors who studied polyolefin catalytic pyrolysis on zeolites of different acidities observed the same qualitative effect.^19,20,74−76^

In addition, the high steam concentration under oxidative conditions has an influence on the plastic cracking mechanism and product distributions. Thus, cracking reactions on the acid sites of the zeolites proceed via the carbonium ion mechanism,^77,78^ but steam cracking proceeds by the free radical mechanism.^79^ Zeolites contain both Brønsted (proton acid sites) and Lewis (nonproton acid sites) sites. Cracking reactions over Brønsted acid sites take place via the carbonium ion mechanism, whereas cracking on Lewis acid sites proceeds via both carbonium ions and free radical mechanisms.^79^ In this respect, both the free radical mechanism and the carbonium ion mechanism play significant roles in the catalytic cracking at high temperatures over zeolites in the presence of steam. Meng et al.^80^ studied the competition between the free radical mechanism and the carbonium ion mechanism in the catalytic steam cracking over different catalysts. They proved that the free radical mechanism has a remarkable role under temperatures of the same order of those used in this study. The aforementioned steam impact on the reaction mechanism also has an influence on products yields. In fact, several studies have proven the positive effect of a steam presence on the catalytic cracking of hydrocarbons over zeolites by significantly promoting the selectivity toward light olefins.^81−84^

It should also be noted that the impact of oxygen (or steam) in the reaction environment is not only restricted to the improvement in catalysts activity, but it may also affect the initial steps of plastic thermal degradation prior to catalytic cracking. Thus, several authors have reported the positive effect of an oxygen presence on plastic pyrolysis, as it promotes thermal degradation at low temperatures, i.e., a faster degradation rate than in the absence of oxygen.^85,86^ Thus, thermal degradation takes place via a radical scission mechanism, and the autocatalytic nature of the polymer oxidation radical mechanism contributes to the overall reaction acceleration.^87^ Therefore, the modification of the reaction mechanism for thermal pyrolysis has also an influence on the results obtained in the catalytic cracking.

#### Gas Product

**Figure 3:**
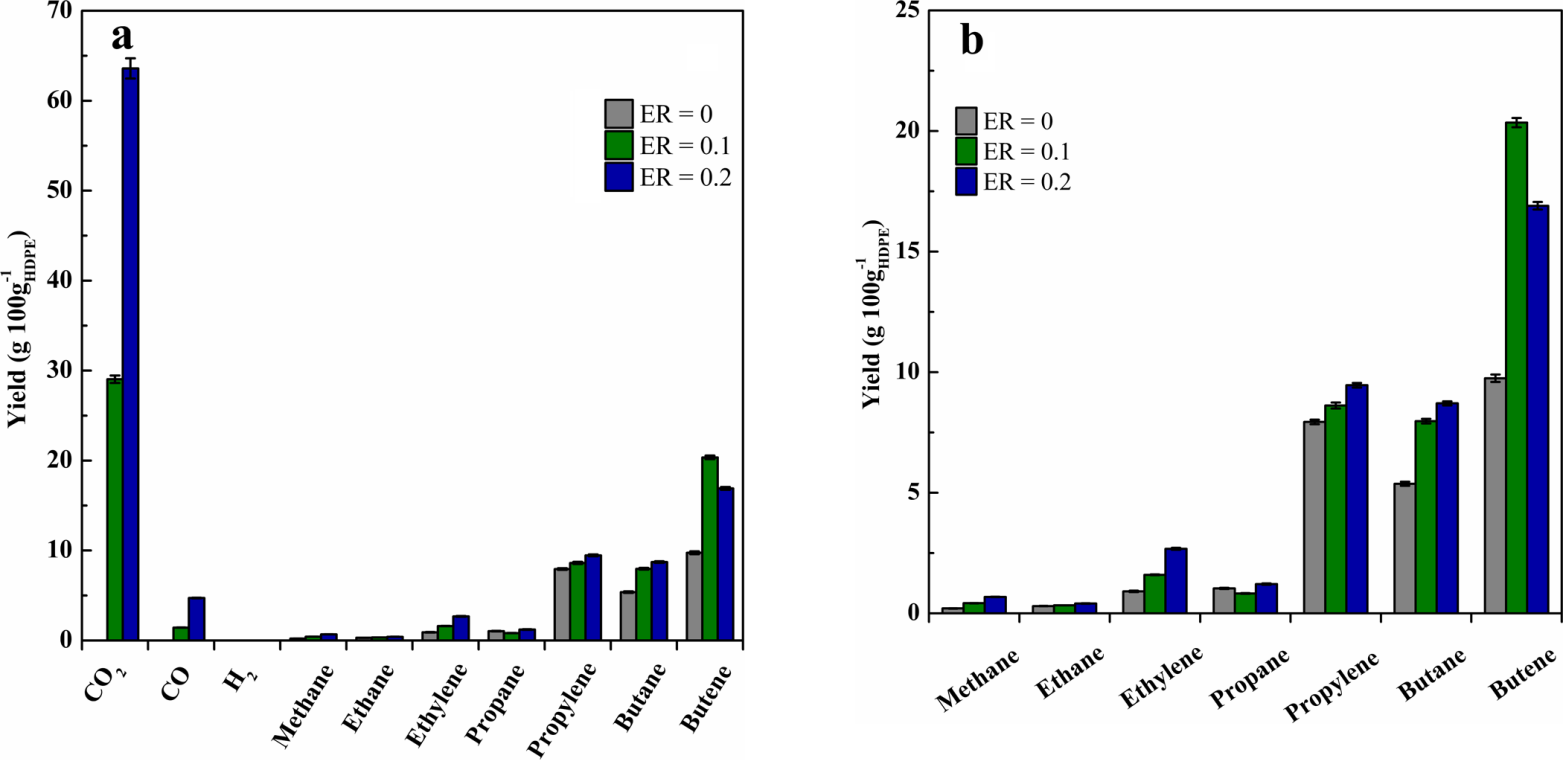
Gas product yields obtained in the oxidative catalytic pyrolysis with different ER ratios at 550 °C on the FCC catalyst. All the products (a) and only hydrocarbons (b).

Figure 3 shows the effect of the ER ratio on gaseous product yields. An increase in ER leads to a dramatic impact on gaseous product yields. Thus, light olefins are the prevailing compounds when no oxygen was in the feed (ER = 0). However, CO_2_ was the main gaseous product under oxidative conditions (Figure 3a). Furthermore, given the relatively low ER values used, CO/CO_2_ ratios were high. Figure 3b shows that ER has a significant influence on the gaseous hydrocarbon yields (CO and CO_2_ yields have not been included for a clearer evaluation of the ER role). An increase in ER caused an increase in the yields of all gaseous hydrocarbons with the exception of butenes, which peaked for an ER value of 0.1. Moreover, interesting conclusions may be drawn concerning the evolution of C_1_–C_4_ hydrocarbons. Thus, the evolution of the ethylene/propylene ratio with ER is especially remarkable, as it increased from a value of 0.11 for ER = 0 to 0.28 for ER = 0.2. This trend may be associated with the higher activities of the catalysts operating under oxidative pyrolysis conditions. Thus, activity enhances olefin oligomerization-cracking reactions involving light olefin interconversion, which increases the final product of this mechanism, i.e., ethylene.^78^ The influence of ER on the individual yields of light alkanes is not as significant as on the yields of olefinic compounds. Nevertheless, a slight increase in the yields of light alkanes was observed when oxygen was in the feed due to the enhancement of cracking and secondary hydrogen transfer reactions.^19^ Finally, the yields of methane and ethane are very low, even when operating with ER = 0.2, which is associated with a low residence time in the conical spouted bed reactor attenuating secondary thermal cracking reactions. Therefore, oxidative catalytic pyrolysis of HDPE on the spent FCC catalyst has proven to be a suitable process for the production of valuable light olefins. In fact, cofeeding oxygen into the pyrolysis reactor greatly improved the production of these chemicals; i.e., their yield was 18.6% under conventional pyrolysis conditions, but reached a value of around 30% when operating with both ER values of 0.1 and 0.2. This result is of great relevance, as oxygen incorporation into catalytic pyrolysis remarkably reduced hydrocarbon yields, and the selectivity toward light olefins increased from 18.6% for ER = 0 to 37.6% for ER = 0.2.

#### Liquid Product

**Figure 4:**
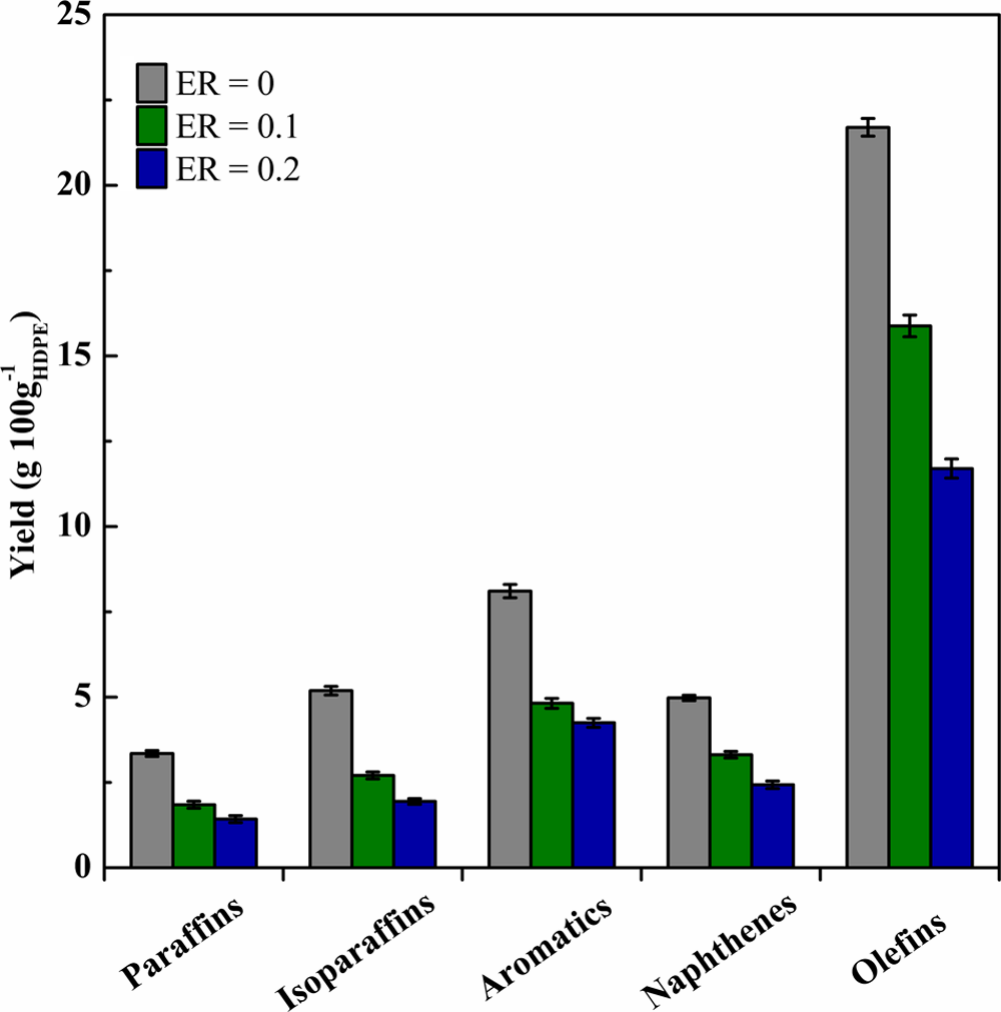
Effect of ER on C_5_–C_11_ compound yields sorted according to their chemical bond.

Operation under oxidative conditions shifted the product distribution of HDPE catalytic pyrolysis from liquid hydrocarbons (the main products obtained in conventional pyrolysis) to gaseous ones. This section deals with the influence ER has on the liquid product composition. Thus, Figure 4 shows the compositions of the C_5_–C_11_ fraction according to their chemical structures. It should be noted that the analysis of the heavy liquid fraction (C_12_–C_18_) by GC-MS is challenging (a considerable fraction of nonidentified compounds), and the results shown in Figure 4 are therefore limited to the light fraction (C_5_–C_11_).

As observed, an increase in ER decreased the yields of all C_5_–C_11_ families, with olefins being the main compounds under all the conditions studied. These olefins are formed in the primary catalytic cracking of the plastic-derived oligomers. However, the limited extent of hydrogenation and cyclization hindered the formation of paraffins and aromatics, respectively. Likewise, other authors also reported that olefins were the prevailing compounds in the liquid fraction, with the aromatic content being low, in the pyrolysis of plastics on spent FCC catalysts due to its moderate acidity.^88,89^

It is also noteworthy that, within the ER range studied (0.0–0.2), this parameter has a rather limited effect on the concentration of each family in the C_5_–C_11_ fraction. Thus, there was only a slight decrease in all the families, except that of olefins, which underwent a moderate decrease as ER is increased.

Considering the compositions of the C_5_–C_11_ fraction, this stream may be incorporated into the refinery gasoline pool after a mild hydrotreatment to reduce the presence of olefins and aromatics.^17^

**Figure 5:**
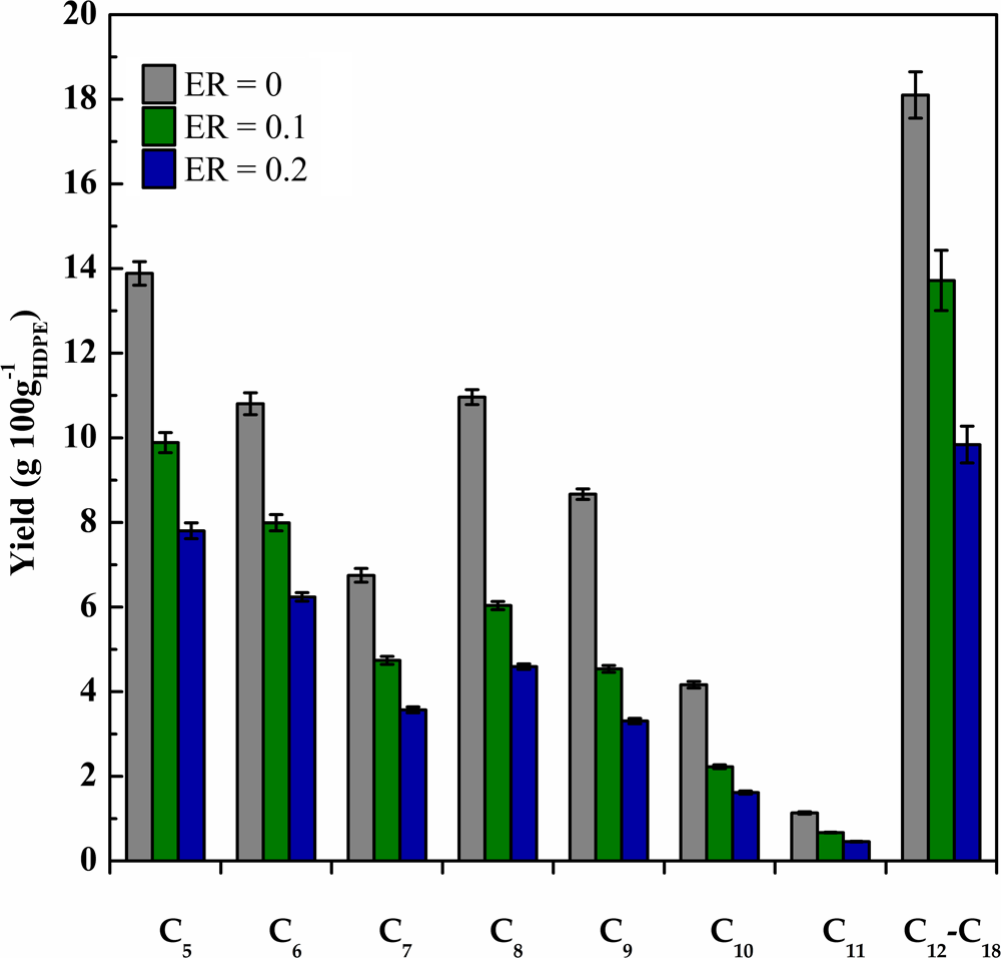
Liquid fraction yields obtained in the catalytic pyrolysis with different ER ratios according to the number of carbon atoms.

Figure 5 shows the evolution of oil products with ER according to their carbon atom numbers. It is noteworthy that the hydrocarbons in the C_5_–C_11_ range are plotted individually, whereas those heavier than C_12_ are grouped together. Due to the suitable cracking activity of FCC catalysts in the conical spouted bed reactor, the presence of products heavier than C_18_ was negligible. As observed in Figure 5, an increase in the oxygen partial pressure in the reactor improved the cracking performance; therefore, all the liquid fractions were reduced due to their cracking to yield gaseous compounds. However, this reduction was more significant for the fraction having between 8 and 11 carbon atoms, whereas those containing from 5 to 7 carbon atoms underwent a lower reduction. Finally, the cracking of the heaviest C_12_–C_18_ fraction was almost proportional to that of the overall pyrolysis oil.

Table 2 shows the detailed oil composition determined by the GC-MS technique. This identification was easier in the lighter fraction, C_5_–C_11_, than in the heavy oil fraction, C_12_–C_18_, and therefore, the number of nonidentified compounds is higher in the latter fraction. As observed, the prevailing compounds in the pyrolysis oil are olefins, especially those of few carbon atom numbers. In all the cases, the yields of paraffins are remarkably lower than those of the corresponding olefins of the same carbon atom numbers. Interestingly, the aromatic compounds detected are benzene derivatives, such as toluene and xylenes. However, no significant formation of PAHs was detected in the heavy fraction. It should be also noted that no oxygenated compounds were detected in the oil, even operating with an ER value of 0.2.

**Table 2:** Detailed Composition of Oil Obtained in Oxidative Catalytic Pyrolysis of HDPE under Different ER Values

	ER = 0	ER = 0.1	ER = 0.2
Compound	g/100 g_HDPE_	g/100 g_HDPE_	g/100 g_HDPE_
Pentenes	8.88	6.82	5.41
Hexane	1.00	0.65	0.49
Hexenes	5.25	4.34	3.03
Benzene	0.51	0.50	0.59
Heptenes	2.96	2.03	1.42
Toluene	1.12	0.92	0.96
Octane	0.11	0.15	0.15
Octenes	2.29	1.49	0.96
Xylenes	1.50	0.86	0.74
Ethyl benzene	2.14	1.01	0.85
Nonane	1.56	0.74	0.57
Nonenes	1.44	0.60	0.41
Propyl benzene	0.42	0.22	0.17
Ethyl methyl benzene	0.82	0.35	0.26
Trimethyl benzene	0.46	0.43	0.29
Decane	0.52	0.12	0.09
Decenes	0.57	0.32	0.24
Diethyl benzene	0.24	0.15	0.08
Methyl propyl benzene	0.25	0.11	0.08
Ethyl dimethyl benzene	0.14	0.27	0.23
Undecane	0.19	0.19	0.13
Undecenes	0.54	0.29	0.22
Isoparaffins	5.24	2.71	1.94
Naphthenes	5.03	3.31	2.43
Non identified	13.20	7.51	5.84
**C**_**5**_**–C**_**11**_	**56.37**	**36.09**	**27.58**
Dodecane	0.43	0.18	0.13
Dodecene	1.00	0.08	0.42
C_12_ not identified	2.65	1.57	0.79
Tridecane	0.48	0.20	0.11
Tridecene	0.40	0.13	0.09
C_13_ not identified	3.08	2.28	1.43
Tetradecane	0.40	0.25	0.17
Tetradecene	0.44	0.14	0.08
C_14_ not identified	2.22	1.46	1.08
Pentadecane	0.39	0.29	0.16
Pentadecene	0.21	0.08	0.16
C_15_ not identified	1.49	0.84	0.80
Hexadecane	0.23	0.61	0.31
C_16_ not identified	1.29	1.54	1.14
Heptadecane	0.13	0.47	0.26
Heptadecene	0.16	0.21	0.21
C_17_ not identified	0.73	1.47	1.16
Octadecane	0.43	0.61	0.28
C_18_ not identified	1.94	1.31	1.06
**C**_**12**_**–C**_**18**_	**18.10**	**13.72**	**9.84**

## Conclusions

Oxidative pyrolysis is an interesting alternative for the full-scale development of waste plastic pyrolysis, as it allows overcoming the technical barriers associated with heat supply to the pyrolysis reactor. An ER value of around 0.15 is required to attain autothermal operation and avoid an external heat supply to the process.

It is noteworthy that the operation under oxidative conditions improved the FCC catalyst performance. Thus, an oxygen presence contributed to in situ combustion of the coke deposited on the FCC catalyst and its partial regeneration, which improved its activity throughout continuous operation. Moreover, the steam generated in combustion reactions also contributes to modifying the reaction mechanism and enhancing cracking reactions.

Furthermore, the higher cracking activities of FCC catalysts under oxidative conditions enhances the conversion of the oil fraction to gaseous products. The high yields of valuable light olefins obtained under oxidative pyrolysis conditions are especially remarkable, as their yields increased from 18.6% for ER = 0 to around 30% when operating with ER values of 0.1 and 0.2. However, the yield of the C_5_–C_11_ fraction decreased from 56.4% operating under inert pyrolysis to 27.6% operating with an ER of 0.2. Similarly, the yield of the heavy oil fraction, C_12_–C_18_, decreased from 18.1 to 9.9 in the same ER range.

These results are encouraging for the full-scale development of the process, as oxidative pyrolysis not only eases heat integration in the pyrolysis process, but also increases reactor throughput due to the higher catalyst activity and selectivity toward valuable chemicals from waste plastics.
